# Improved Growth Patterns in Cystic Fibrosis Mice after Loss of Histone Deacetylase 6

**DOI:** 10.1038/s41598-017-03931-2

**Published:** 2017-06-16

**Authors:** Sharon M. Rymut, Deborah A. Corey, Dana M. Valerio, Bernadette O. Erokwu, Chris A. Flask, Thomas J. Kelley, Craig A. Hodges

**Affiliations:** 1 Department of Pediatrics, Case Western Reserve University, Cleveland, Ohio USA; 2 Department of Radiology, Case Western Reserve University, Cleveland, OH USA; 3 Department of Biomedical Engineering, Case Western Reserve University, Cleveland, OH USA; 4 Department of Genetics and Genome Sciences, Case Western Reserve University, Cleveland, OH USA

## Abstract

Growth failure in cystic fibrosis (CF) patients has been well-documented and shown to correlate with poorer disease outcomes. This observation is also true in CF animal models, including mouse, pig, rat, and ferret. The etiology underlying growth deficits is unknown, and our previous work demonstrated reduced tubulin acetylation in CF cell models and tissue that is correctable by inhibition of histone deacetylase-6 (HDAC6). Here, we hypothesize that loss of HDAC6 will improve growth phenotype in a CF mouse model. *Hdac6* knockout mice were crossed with *F508del* (CF) mice to generate *F508del*/*Hdac6* (CF/HDA) mice. Growth, fat deposits, survival, and bioelectric measurements were analyzed. *CF*/*HDA* mice displayed improvements in length and weight with no correction of CFTR function. Mechanistically, *Igf1* levels likely account for increased length and improvements in fertility. Weight gain is attributed to increased fat deposits potentially mediated by increased adipocyte differentiation. CF-related growth deficits can be improved via inhibition of HDAC6, further implicating it as a potential therapeutic target for CF.

## Introduction

Cystic fibrosis is an autosomal recessive disorder caused by mutations in the cystic fibrosis transmembrane conductance regulator (CFTR). Although pulmonary disease is the primary cause of mortality, CF is a multisystem disorder with manifestations in the reproductive system, sweat ducts, and digestive systems among others. One phenotype associated with CF is growth impairment, characterized by both reduced height and weight^1, 2^. Growth impairment is significant clinically as lung function for CF patients has been directly correlated with body mass index (BMI)^3–6^. Also, growth impairment is a consistent phenotype observed in multiple CF animal models, including the ferret, pig, rat, and mouse models^7–10^. Mechanistically, it has been proposed that poor nutrient absorption and reduced gastrointestinal pH may contribute to growth impairment. However, we have demonstrated that impaired growth in the CF mouse is unrelated to CFTR function in the intestinal epithelium^11^. Another mechanism proposed to explain impaired growth in CF is reduced levels of IGF-1. Rosenburg *et al*. have demonstrated in CF mice that IGF-1 levels are reduced compared to control mice^7^, a finding that has been repeated in the CF pig model^8^. These findings of reduced IGF-1 levels in CF are also consistent with clinical data demonstrating similar reduction of IGF-1 in patients with CF^12^.

Given the potential clinical importance of improving growth, growth hormone therapies have been attempted in CF trials. A limited clinical trial in 2001 hypothesized synthetic IGF-1 would improve growth of CF patients aged 9–13; however, no significant improvements were observed^13^. The efficacy of treating CF patients with recombinant growth hormone has also been examined. A meta-analysis of studies treating CF patients with recombinant growth hormone concluded that height, weight, and lean body mass are improved compared to untreated controls. However, no long-term benefits in quality of life or health status were observed^14^. Recently, it was noted that ivacaftor used for patients with the G551D mutation improved CF patients’ weights and body mass index z-scores; however, little improvement has been noticed for heights^15^. Current long-term studies for CFTR corrector therapies (i.e. ivacaftor and lumacaftor) are yet to be completed and may highlight new evidence supporting CFTR modulators to be beneficial for overcoming growth defects in CF patients. Although there is promise that correcting CFTR function will be beneficial in improving growth, there is no clear understanding of the mechanisms of growth deficiency in CF and no distinctive therapeutic targets to address growth, especially in those patients with genotypes not currently amenable to pharmacological correction of CFTR function.

Recently, we have identified that CF cells exhibit significant alterations to microtubule regulation. One of those alterations is reduced levels of acetylated tubulin in CF cells compared to non-CF controls. Histone deacetylase-6 (HDAC6) is a cytoplasmic deacetylase important in transcriptional regulation and has over ten binding partners, most notably alpha-tubulin^16^. With HDAC6 as a key modulator for tubulin acetylation, HDAC6 inhibition has been shown to restore tubulin acetylation to normal levels in CF cells. Further, inhibition of HDAC6 in CF cells restores intracellular transport defects and reduces inflammatory signaling^16^. We have developed a double KO mouse model by crossing *F508del Cftr* mice with *Hdac6-*null mice (*F508del*/*Hdac6*) to determine the *in vivo* effects of restoring microtubule acetylation. *F508del*/*Hdac6* mice are viable and have an increase in tubulin acetylation compared to *F508del* mice. Increased acetylated tubulin of the *F508del*/*Hdac6* mice did not improve CFTR function as determined by nasal potential differences. Interestingly, both lengths and weights of *F508del*/*Hdac6* mice improve to that of wild-type (WT) control. Improvements in lengths can be attributed to increased *Igf1* levels, while increased inguinal fat contribute to improvements in weight. This manuscript is the first report of a correction of CF-related growth defects and points to microtubule acetylation as a novel therapeutic target to improve growth in CF patients.

## Results

### Generation of CF/HDA mice

**Figure 1:**
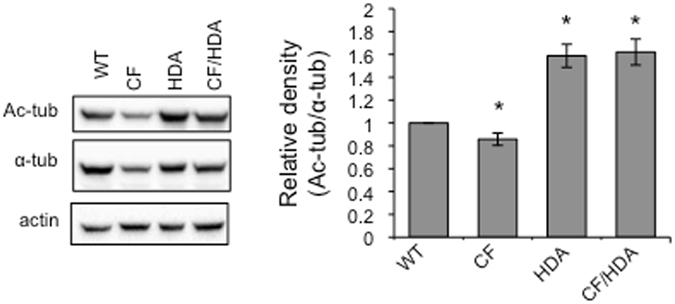
Increased microtubule acetylation in *CF*/*HDA* mice. Nasal epithelium from wild-type (WT), *F508del* (*CF*), *Hdac6*−/− (HDA), and *F508del*/*Hdac6* (CF/HDA) were excised, lysed, and analyzed via Western blot. Representative blots of 17 experiments are shown for acetylated-α-tubulin (ac-tub), α-tubulin, and actin (left). Gels are cropped for space. Full-length gels are provided in Supplemental Data. Quantification of ac-tub normalized to α-tubulin is shown (right). Significance between all groups was determined (*p < 0.05; *n* = 17; 1-way ANOVA with Newman-Keuls multiple comparison post hoc test). Data represent the means ± S.E.M.

Increasing microtubule acetylation in CF epithelial cells resulted in decreased NF-κB activation as well as increased endosomal transport, indicating the importance of microtubules in normalizing CF cell biology to wild-type (WT) patterns^16^. To better analyze the *in vivo* impact of microtubule regulation in CF, *Hdac6* was depleted in a CF mouse model to determine the impact of chronic HDAC6 inhibition on CF phenotypes. The mice were developed by crossing HDAC6-null mice (HDA) with *F508del* (CF) heterozygous mice to obtain *F508del*/*Hdac6* double mutant mice (CF/HDA). The *F508del Cftr* mutation was chosen for these studies since it is the most prevalent CFTR mutation and the *F508del* mouse model parallels many CF disease presentations, including reduced growth^17^. Analyzing the nasal epithelium in the CF/HDA mice, results confirm that the genetic knockout of *Hdac6* leads to increased acetylated-alpha-tubulin (ac-tub) expression (Fig. 1). Reduced ac-tub in CF mouse nasal epithelium is consistent with our previous results, though total tubulin appears to be lower in some CF samples than we found in *Cftr*-null (S489X/S489X) mice in our previous study^16^.

**Figure 2:**
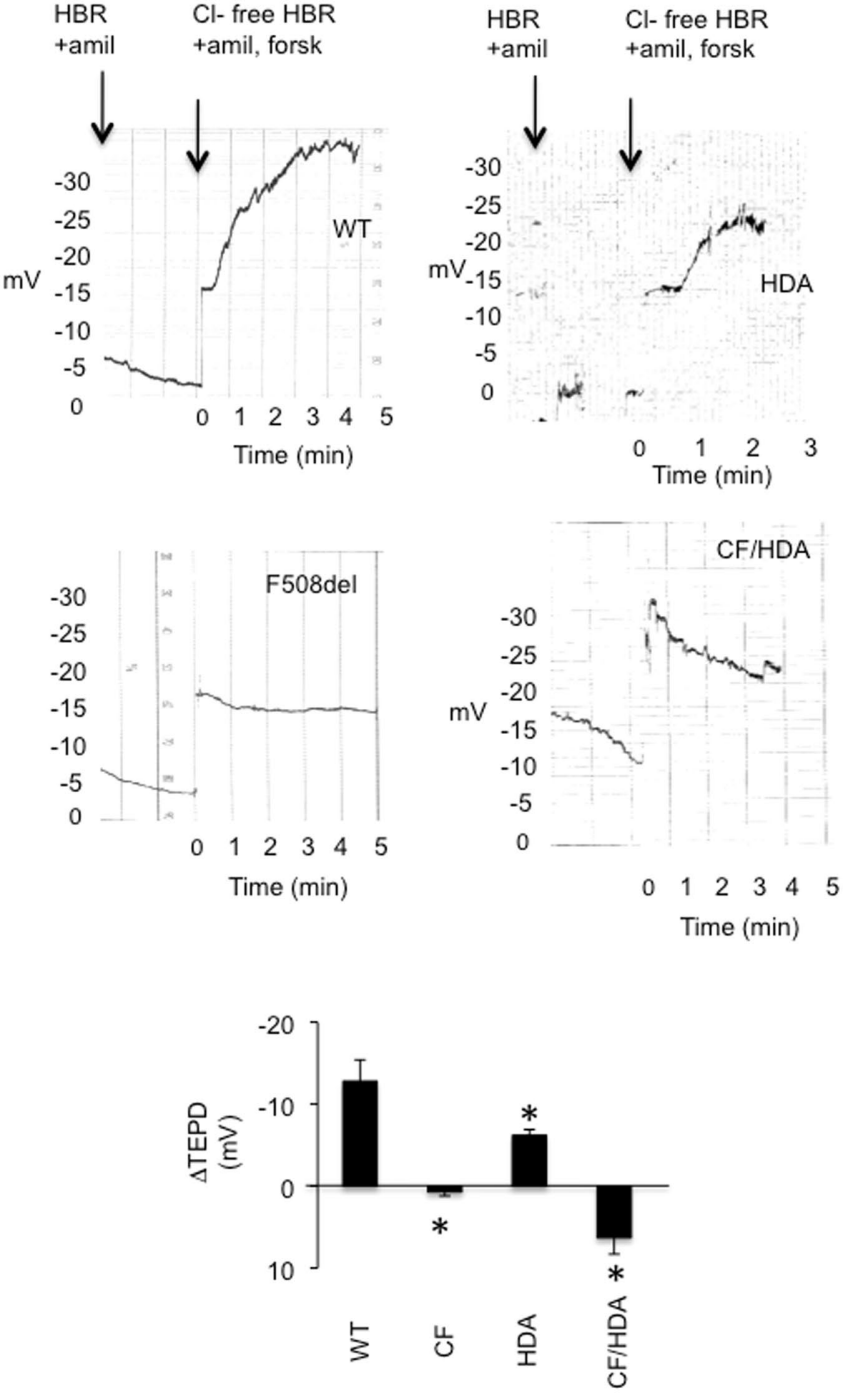
Loss of HDAC6 does not improve CFTR function in *CF*/*HDA* mice. Nasal potential difference (NPD) traces for wild type (WT), F508del (CF), *Hdac6*−/− (HDA) and *F508del*/*Hdac6* (CF/HDA) mice. Mice are perfused initially with HEPES-buffered Ringer’s (HBR) in the presence of amiloride (amil; 100 µM), and then perfusion is transitioned to Cl^−^-free HBR containing amil and forskolin (forsk; 10 µM). Graph shows averaged change in transepithelial potential difference (ΔTEPD) in response to Cl^−^-free HBR/forsk. Significance between all groups was determined; WT (n = 4), HDA (n = 4), CF/HDA (*n* = 4) and *CF* (*n* = 3). Significance determined by 1-way ANOVA with Newman-Keuls multiple comparison post hoc test. Significance compared to WT response is shown (*p < 0.05). Data represent the means ± S.E.M.

Hutt *et al*. have reported that HDAC6 inhibition can increase F508del CFTR processing due to interference with autophagy, resulting in more F508del CFTR being localized to the plasma membrane^17^. To determine whether *Hdac6* depletion influences F508del function in the mouse model, CFTR function was analyzed by nasal potential difference (NPD). Chloride efflux was stimulated by the perfusion of forskolin (10 µM) and amiloride (10 µM) to activate CFTR in chloride-free Ringer’s solution to drive chloride secretion. If there is a lack of functional CFTR in the mice, then little to no change in potential difference will be recorded. Perfusion of chloride-free buffer with forskolin/amiloride in WT mice stimulates a hyperpolarization of −12.3 ± 3.1 mV (Fig. 2). HDA mice also show a normal response to chloride-free Ringer’s and forskolin/IBMX demonstrating that Hdac6 depletion has no functional impact on CFTR function. If anything, HDA mice have slightly reduced chloride efflux compared to WT mice. It is important to note, however, that the nasal potential difference assay is largely a qualitative assay showing the presence or absence of CFTR response. There is sufficient variability in the assay to make quantitative conclusions aside from the presence or absence of CFTR response difficult. CF/HDA mice act just as CF mice showing no hyperpolarization under the same conditions (Fig. 2; p < 0.01; 3–4 mice per genotype). These results demonstrate that depletion of *Hdac6* has no appreciable effect on F508del CFTR activation in this mouse model.

### CF/HDA mice display improved growth patterns compared to CF mice

**Figure 3:**
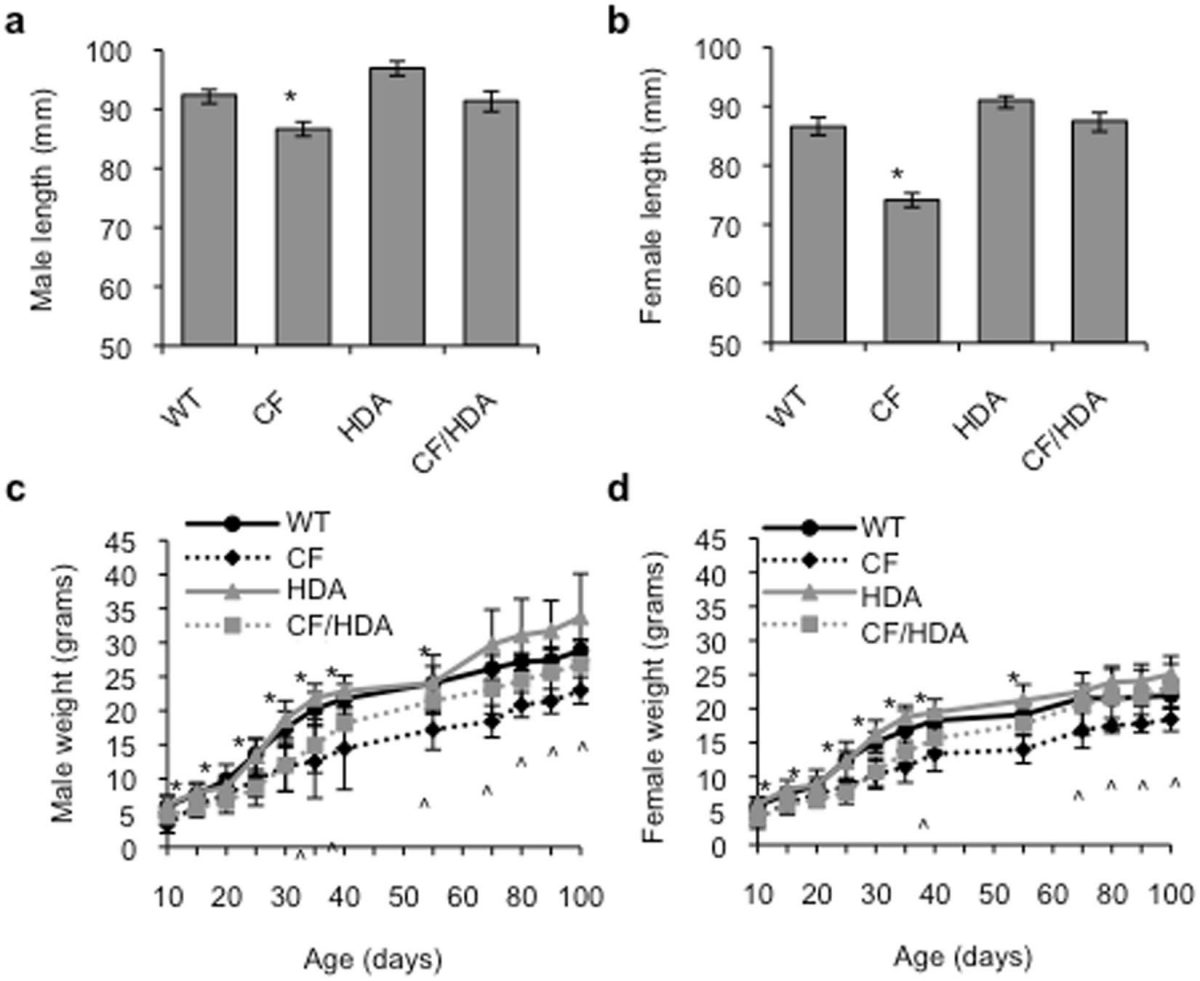
Improved growth phenotype in CF after loss of HDAC6. (**a,b**) Lengths of 8–10 week old wild-type (WT), *F508del* (*CF*), *Hdac6*−/− (*HDA*), and *F508del*/*Hdac6*−/− (CF/HDA) (**a**) male mice and (**b**) female mice (*p = 0.001 for CF vs. all other groups; no other comparisons are significant (p > 0.05); 1-way ANOVA with post-hoc Newman-Keuls test; *n* = 5–11 mice). (**c**,**d**) Weight curves of 8–10 week old WT, *CF*, *HDA*, and CF/HDA (**a**) male and (**b**) female mice (*p < 0.05 for WT v CF/HDA; ^p < 0.05 for CF v CF/HDA; *n* = 12–48 mice for days 0–40; n = 6 for days 40+). Data represent the means ± standard deviation.

A well-characterized phenotype of CF animal models is impaired growth^7, 8^. Growth defects in the mouse have been well studied and shown to be unrelated to intestinal CFTR function or to changes in fat absorption^11, 18^. To analyze growth patterns of CF/HDA mice, both length and weight were observed over time. Consistent with previous reports, CF mice were shorter than WT counterparts for both females and males at 55–60 days (Fig. 3a,b). Female WT mice were 86.6 ± 1.6 mm in length compared to CF mice that are only 74.1 ± 1.2 mm in length (Fig. 3b, p = 0.001; n = 7–9). Likewise, CF male mice (86.6 ± 1.2 mm) are shorter than WT controls (92.4 ± 0.9 mm) (Fig. 3a, p = 0.001, n = 5–11). Deletion of Hdac6 increased the length of CF female mice to 88.5 ± 1.9 mm, an average increase of 14.4 mm over CF mouse length and 1.9 mm longer that WT controls (p = 0.001, n = 7–10). Male CF/HDA show a similar increase in length 91.1 ± 3.6 mm, an increase of 4.5 mm over CF mice (p = 0.001, n = 5–11). These data are significant because they represent the first reported correction of CF-related growth impairment.

Another aspect of growth that is decreased in CF is weight. Regardless of gender, CF and CF/HDA weight gain was identical from day 10–30. After day 30, weights of the CF/HDA female mice increased significantly compared to CF, where CF/HDA mice were 18.4% heavier at day 35 (CF/HDA: 13.54 ± 1.62 g vs. CF: 11.44 ± 2.30 g, p < 0.05, n = 12–15) and remained 26.5% heavier throughout adulthood (Fig. 3d). Comparing female early adulthood (day 55), CF/HDA mice remained smaller than WT mice (CF/HDA: 17.70 ± 1.57 g vs. WT: 19.09 ± 1.54 g, p < 0.05, n = 15–48), however, the difference between these groups is not statistically significant and disappears in late adulthood (day 70–90) (Fig. 3d, p > 0.05, n = 6). These trends also apply to the weights of male CF/HDA mice, where CF/HDA mice were 12.5% heavier at day 35 (CF/HDA: 14.09 ± 4.02 g vs. CF: 12.52 ± 5.36 g, p < 0.05, n = 14–15) and increased the difference by 12% by day 55 (CF/HDA: 21.42 ± 1.97 g and CF: 17.22 ± 2.95 g, p < 0.05, n = 14–15) (Fig. 3c).

### Hdac6 depletion significantly increases fat deposition

**Figure 4:**
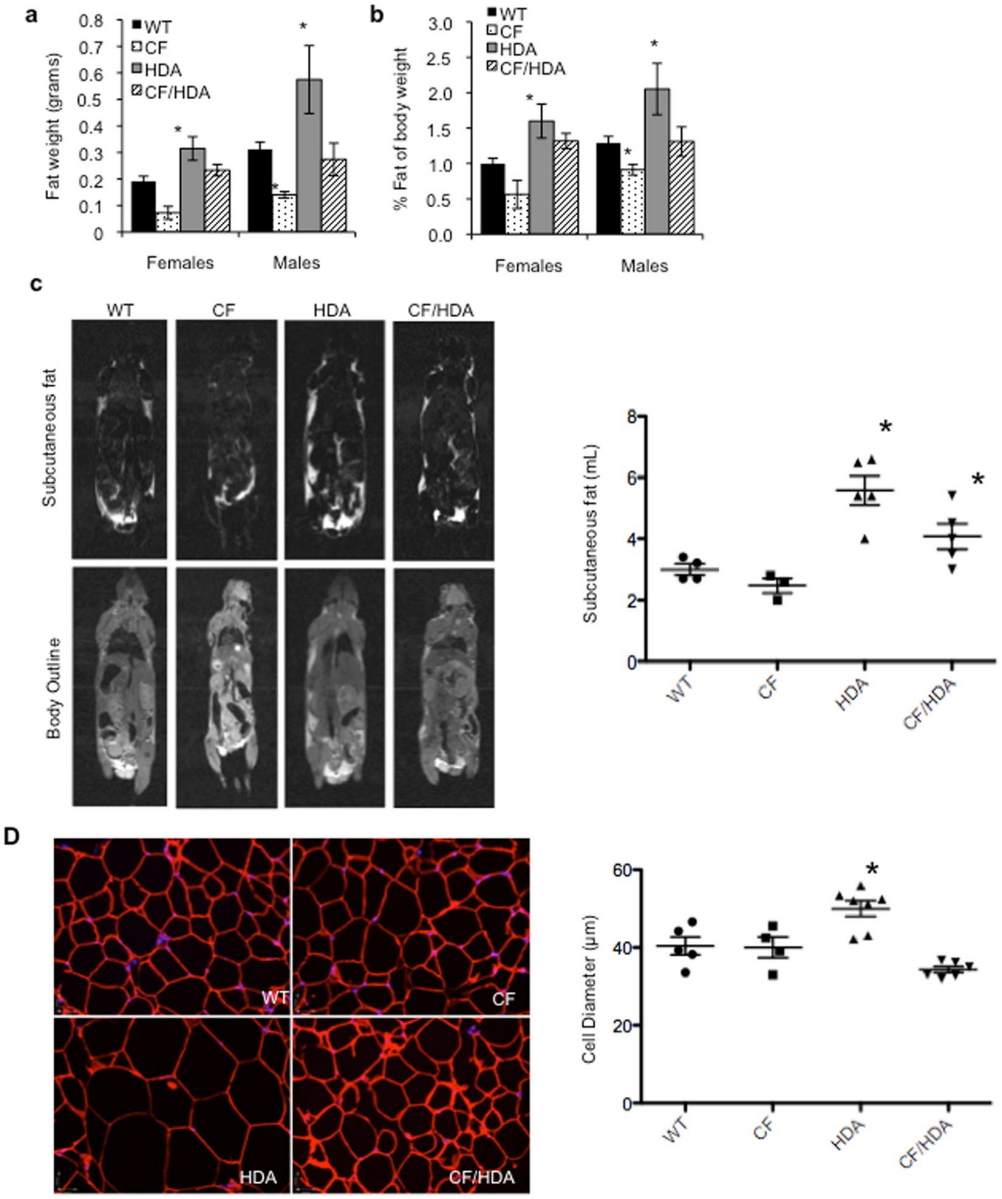
Higher fat depositions in CF/HDA mice compared to WT mice. (a) Inguinal fat was excised and quantified from 8–10 week old wild-type (WT), *F508del* (*CF*), *Hdac6*−/− (*HDA*), and *F508del*/*Hdac6*−/− (CF/HDA) mice (*p < 0.05; *n* ≥ 5). Data represent the means ± S.E.M. (**b**) Percent of inguinal fat to total body weight in 8–10 week old WT, *CF*, *HDA*, and CF/HDA male and female mice (*p < 0.05; *n* ≥ 5). Data represent the means ± S.E.M. (**c**) *In vivo* measurement of adipose tissue depots in male mice via MRI. Representative images are displayed along with quantification of subcutaneous fat volume of individual mice tested. (*p < 0.05 compared to HDA v WT and CF/HDA v. CF by ANOVA with Newman-Keuls multiple comparison test). (**d**) Immunohistochemistry was performed on excised inguinal fat from 8–15 week old mice. Perilipn A denotes adipocyte cell periphery and DAPI stain is used to identify nuclei. Representative images are displayed along with quantification of average cell diameter of each individual mouse tested. (*p < 0.05 compared to HDA v WT by ANOVA with Newman-Keuls multiple comparison test).

Decreased weights in CF can be attributed to decreased heights or lengths, but also to less fat deposition on both patients and animals, respectively. We analyzed next the fat deposition of CF/HDA mice, hypothesizing the deposition as a large contributor to the observed increased mice weights. Inguinal subcutaneous fat depots are one of the earliest fat deposits to form in rodents and can be easily excised for measurements. As expected, a 55.0% decrease in excised fat weight was observed in CF males, while CF females exhibited a 62.1% decrease compared to WT controls (Fig. 4a). Consistent with the above weight measurements, there were higher fat depositions in both HDA and CF/HDA mice regardless of gender. Highlighting the females, CF/HDA inguinal fat was significantly higher than WT mice (0.23 ± 0.02 g vs 0.19 ± 0.02 g); however, HDA mice fat deposition was nearly twice that seen in WT mice. Normalizing inguinal fat to total body weight, the percent fat remained low in CF mice, while percent fat significantly increased when HDAC6 expression is not present (Fig. 4b).

Changes in body fat elicited by HDAC6 depletion can also be visualized by small animal magnetic resonance imaging (MRI). HDA mice and CF/HDA mice have increased subcutaneous, visceral, and total body adipose tissue volume compared to respective WT and CF controls (Fig. 4c). Subcutaneous fat volumes from individual mice tested are shown.

Since there is clearly more fat in HDA and CF/HDA mice compared to WT and CF mice, histology on inguinal adipose tissue was performed to determine if there were any indications of increased cell number of cell volume in response to HDAC6 depletion. There were no obvious histological differences between WT, CF, or CF/HDA fat samples (Fig. 4d). Interestingly, samples from HDA mice show a clear increase in cell diameter suggesting increased fat accumulation (Fig. 4d). The loss of CFTR function apparently prevents this increase in cell volume by an unknown mechanism. In CF/HDA mice, there is no increase in either cell volume or number so another mechanism must account for improved fat deposition in these mice compared to CF mice. One possibility is that CF pre-adipocytes are less efficient in differentiating into adipocytes, thus preventing fat deposition.

**Figure 5:**
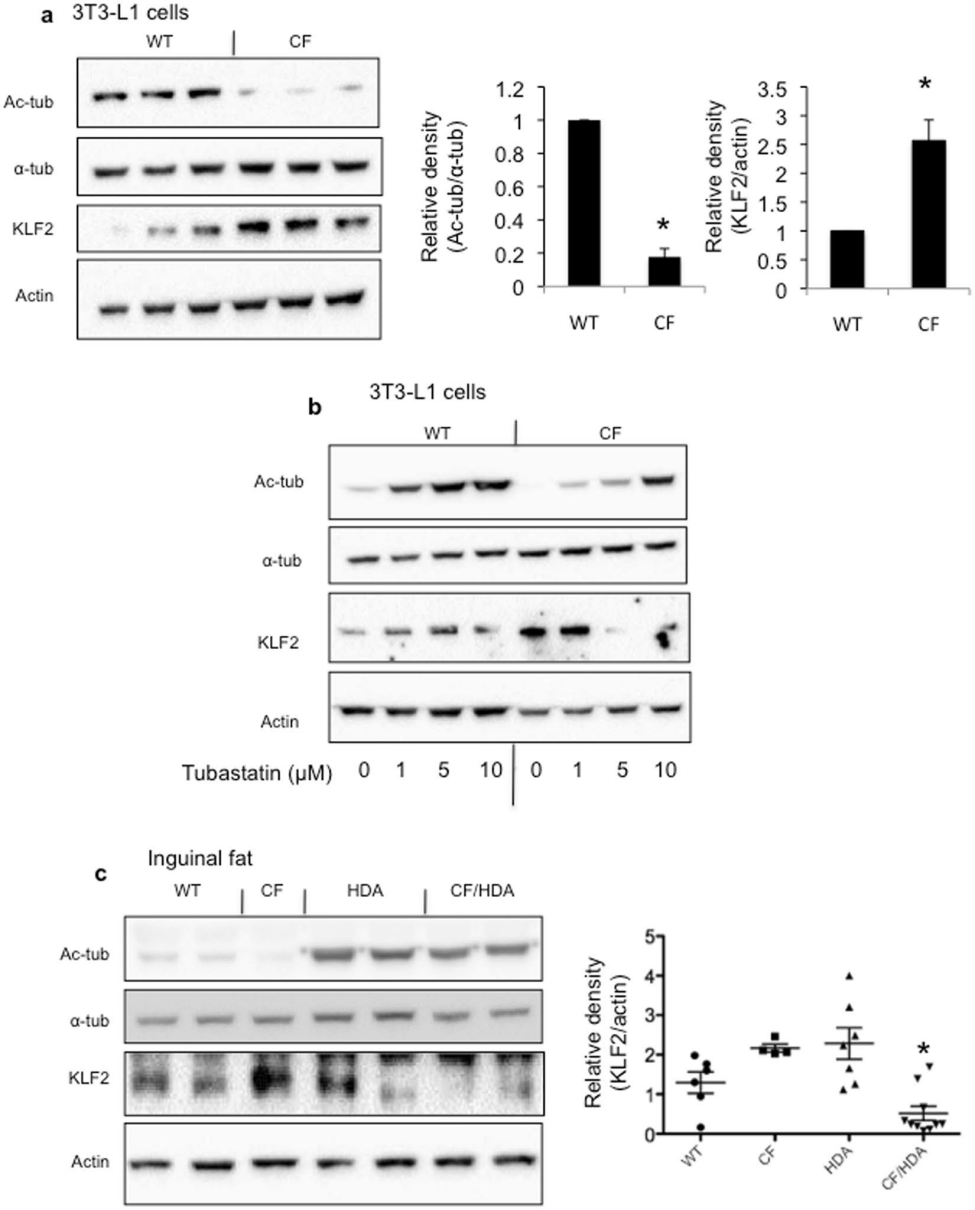
Increased KLF2 expression in CF cultured pre-adipocytes and primary inguinal fat. (**a**) Pre-adipocyte 3T3-L1 cells with CFTR expression ablated (CF) as described in Methods exhibit significantly decreased acetylated α-tubulin (Ac-tub) and increased KLF2 compared to control (WT) cells from each individual mouse tested. Significance between groups was determined by t-test (*p < 0.001; *n* = 6 for Ac-tub; p = 0.001; n = 9 for KLF2); (**b**) Representative gel showing response of Ac-tub and KLF2 expression in WT and CF 3T3-L1 cells to doses of HDAC6 inhibitor tubastatin. Representative of duplicate experiments. (**c**) KLF2 expression in inguinal fat from WT, CF, HDA, and CF/HDA mice. Representative gels showing ac-tub and KLF2 expression is shown. At right, a scatter plot shows values from each individual mouse tested (*p < 0.05 compared to CF v. CF/HDA by ANOVA with Newman-Keuls multiple comparison test). Gels are cropped for space. Full-length gels are provided in Supplemental Data.

To begin testing this hypothesis, 3T3-L1 cells that have been edited to express F508del CFTR (CF) or controls (WT) were examined for ac-tub content and Kruppel-like factor 2 (KLF2) protein expression. High KLF2 expression is known to hinder pre-adipocyte differentiation^19^. CF 3T3-L1 cells exhibit dramatically reduced ac-tub content (approximately 80% reduction) and a 2.5-fold increase in KLF2 expression (Fig. 5a). To determine if these expression levels were sensitive to HDAC6 inhibition, ac-tub and KLF2 expression levels were monitored in WT and CF 3T3-L1 cells in response to various doses of the HDAC6 inhibitor tubastatin (Fig. 5b). Tubastatin leads to a significant increase in ac-tub content suggesting pre-adipocytes are very responsive to HDAC6 inhibitors. KLF2 expression also decreases in CF 3T3-L1 cells, but not in WT cells indicating a CF-specific alteration in KLF2 regulation.

To determine if a similar pattern is observed *in vivo*, inguinal fat was excised from WT, CF, HDA, and CF/HDA mice was examined for ac-tub and KLF2 expression levels. Like 3T3-L1 cells, primary mouse adipose tissue responded vigorously to HDAC6 depletion with a significant increase in ac-tub content (10.1 ± 2.6 fold increase for HDA mice and 11.1 ± 2.5 fold increase for CF/HDA mice compared to WT; p < 0.05 for each by ANOVA with Newman-Keuls multiple comparison test). Likewise, KLF2 levels decreased significantly in CF/HDA adipose tissue, while HDA mouse KLF2 levels were widely divergent (Fig. 5c). Similar to the 3T3-L1 cells model, these data suggest that a CF-specific regulatory event is modulating KLF2 levels and the KLF2 expression is very sensitive to HDAC6 inhibition. Ac-tub levels trended lower and KLF2 levels trended higher in CF adipose tissue as observed in WT and CF 3T3-L1 cells, but neither was significant compared to WT values. It is hypothesized that the adipose tissue is differentiated thus dampening the differences observed in a pre-adipocyte model. These data suggest the possibility that HDAC6 depletion improves adipocyte differentiation in CF mice leading to improved fat deposition. This mechanism needs to be further explored.

### Mechanism underlying growth improvements include elevated Igf1 expression

**Figure 6:**
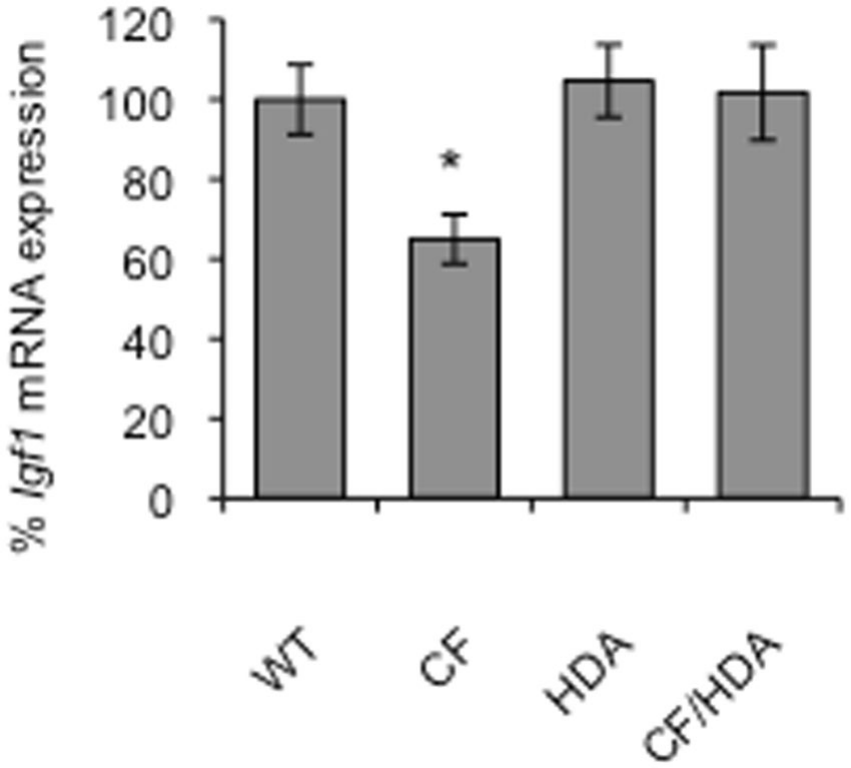
*Igf1* expression is normalized in *F508del*/*Hdac6*−/− mice. Expression of *Igf1* in WT, *F508del* (*CF*), *Hdac6*−/− (*HDA*), and *F508del*/*Hdac6* (CF/HDA) mice. (*p < 0.05 for CF vs. all other groups; no other comparisons are significant; ANOVA with post-hoc Newman-Keuls test; *n* = 5). Data represent the means ± S.E.M.

Though direct effects of HDAC6 depletion on adipose tissue may be partially responsible for weight gain and increased fat accumulation, these effects do not explain improved linear growth. Previous studies have identified that reduced levels of IGF-1 are likely responsible for reduced growth in CF animal models^7, 8^. To determine if increased growth in CF/HDA mice correlates with IGF-1 expression, *Igf1* mRNA levels were determined from liver. Expression of *Igf1* mRNA was detected in all mice, but it was lowest in CF mice consistent with other studies^7^. *Igf1* mRNA levels in CF/HDA were significantly increased compared to CF mice and normalized to WT levels at 55–60 days of age (Fig. 6, p < 0.05, n = 5). These data suggest that increased growth in CF/HDA may be due to restored levels of IGF-1.

IGF-1 production in the liver is controlled by the hypothalamus-pituitary (HP) axis^20, 21^. The HP axis is also a key regulator of reproductive hormone production, which is known to be abnormal in CF female mice and contributes to the infertility^22^. Aspects of the CF female reproductive tract can be normalized with the addition of exogenous hormones. To test whether CF female mouse fertility is impacted by HDAC6 depletion, 6 breeding pairs of CF/HDA mice were established. Five of the six breeding pairs became pregnant and had pups in the first two months of mating compared to one of nine CF female mice in that same time period (p < 0.05). These data further suggest that HDAC6 depletion is restoring HP axis function.

## Discussion

Several studies of growth patterns in CF have demonstrated that CF patients on average are shorter in stature and have less fat deposits than aged matched controls^1, 2^. These growth characteristics have been observed in multiple animal models of CF, including the mouse, pig, rat, and ferret models^7–10^. No interventions or genetic manipulations have been previously identified to improve growth in a CF model. In this study, we demonstrate that depleting HDAC6 expression in an *F508del* mouse model results in mice that are consistently longer and heavier than CF controls. These data suggest that microtubule function is a key regulator of growth and fat storage in CF.

Our previous studies have demonstrated two microtubule changes characteristic in CF cells: 1) reduced tubulin acetylation and 2) reduced rates of microtubule formation. Reduced tubulin acetylation can be corrected by inhibiting the function of the tubulin deacetylase HDAC6. We have shown that HDAC6 inhibition in CF cells corrects many CF cellular phenotypes, including aberrant intracellular transport and increased inflammatory signaling^16^. We also demonstrated that mimicking CF-related changes to microtubule regulation by depleting the expression of the tubulin proliferation promoting protein (TPPP) in WT cells replicates CF cellular phenotypes. TPPP promotes microtubule formation rates and acts as an HDAC6 inhibitor, thus controlling both CF-related microtubule changes we have identified. These data demonstrate directly the importance of microtubule regulation in CF cell biology^23^. It would follow that if microtubule function is key to growth regulation in CF as we propose in this manuscript, then depletion of TPPP expression in a mouse model should mimic CF growth patterns. Based on data from the International Mouse Phenotyping Consortium, TPPP knock-out mice exhibit both the reduced body length and reduced body mass that are characteristic of CF mice^24^. Together with our findings, these data suggest that growth regulation in CF is directly influenced by identified alterations in microtubule regulation. However, a role for other HDAC6 targets mediating changes in growth phenotypes cannot be ruled out and should be considered in future studies.

In addition to improved growth, CF/HDA mice show improved fertility compared to control CF mice. Improvement in both growth and fertility suggest a change in the hypothalamus-pituitary axis in response to HDAC6 depletion. Consistent with this hypothesis, Igf1 levels are increased in the CF/HDA mice. Reduced IGF-1 levels in CF mouse and pig models have been postulated as a direct reason for reduced growth in CF^7, 8^. Human data also demonstrate reduced IGF-1 levels, suggesting that these alterations are a conserved effect of lost CFTR function. Direct analysis reveals less tubulin and reduced ac-tub levels in the hypothalamus of CF mouse and the restoration of these levels in the HDAC6-depleted CF mice. These data demonstrate that CFTR function likely has neurological impact and that growth regulation is influenced by these interactions.

Important to note is that not all CF phenotypes were corrected in the CF/HDA mouse. Survival was unchanged, where 41.3% of F508del mice and 40.6% of CF/HDA mice have died by 40 days of age. Consistent with their CF counterparts, the majority of deaths resulted from intestinal obstruction, indicating that HDAC6 has no implications on the gastrointestinal complications that are often observed in CF animals and patients.

Further elucidation of the mechanisms underlying the weight gain demonstrated in the CF/HDA mice needs to be pursued. One possible mechanism is a direct impact of microtubule acetylation on adipose tissue. Previously, Yang *et al*. demonstrated that cytoskeletal remodeling is a prerequisite for the differentiation of pre-adipocytes to mature adipocytes. The authors conclude katanin, an important microtubule associated protein (MAP), is crucial in the differentiation process^25^. Studies by Forcioli-Conti and colleagues corroborate this work and show the importance of HDAC6. The importance of HDAC6 in regulating the cilium in this process is shown, but mechanisms involving other HDAC6 targets and interacting proteins cannot be ruled out^26, 27^. Our data support the possible involvement of improved adipocyte differentiation in the absence of HDAC6 as a means for increased fat deposition and weight gain in CF/HDA mice. CF 3T3-L1 pre-adipocytes show dramatic decreases in ac-tub levels and an increase in the differentiation regulator KLF2 compared to WT cells. These levels in CF 3T3-L1 cells are reversible in the presence of the HDAC6 inhibitor tubastatin, as well as in primary inguinal adipose tissue in CF/HDA mice. However, KLF2 levels are unchanged in WT cells and tissues in response to HDAC6 inhibition. It is intriguing that weight-gain and increased fat content of HDA mice is likely due to increased lipid storage as fat cell size as determined by histology is significantly increased, but cell size is not altered in CF/HDA mice. These data further point to a CF-specific alteration in adipocyte differentiation as a possible mechanism of reduced weight and fat content in CF mice. The cellular mechanisms of these interactions need to be further elucidated. For example, it is unknown whether KLF2 is simply a marker of altered differentiation regulation in CF pre-adipocytes or a causative agent.

In this study, the *in vivo* importance of HDAC6 inhibition in regulating CF phenotypes is demonstrated. We have previously shown how HDAC6 can influence CF regulation in cell models, but here HDAC6 depletion improves fertility and growth patterns in CF mice, including both significant gains in length and weight. Two contributing mechanisms have been identified. Improved IGF-1 levels likely contribute to fertility restoration and linear growth, while HDAC6 inhibition appears to have direct impact on adipocyte cells, perhaps facilitating pre-adipocyte differentiation in CF samples. Combined, these studies provide important insight into growth regulation in CF and suggest that targeting HDAC6 function with now available small molecule inhibitors may prove to be a viable approach to address growth issues in CF.

## Methods

### Mice

To create mice without functional CFTR and HDAC6 we crossed mice with the *Cftr* mutation *F508del* ^28^ with HDAC6 null mice^29^. Both strains were on a C57Bl/6 J background. To decrease the incidence of intestinal obstruction that is common in CF mice, mice were allowed access to sterile water with osmotic laxative, PEG-3350 with electrolytes (Kremers Urban). All mice were maintained on a 12 h light, 12 h dark cycle at a mean ambient temperature of 22 °C. The Institutional Animal Care and Use Committee (IACUC) of Case Western Reserve University approved all animal protocols. All methods were conducted according to necessary guidelines and established regulations.

### 3T3-L1 cells

3T3-L1 cells (ATCC) were grown in DMEM, 10% calf serum, and 1% penicillin-streptomycin, amphotericin B. 3T3-L1 cells were transfected with pCas9-GFP (Addgene), mouse CRISPR exon 11 (Invitrogen GeneArt), and template plasmid pCR2.1TOPOdeltaF508 at a 2:1:1 ratio with 60 ul of Lipofectamine 2000 (Invitrogen) per confluent 10 cm dish. After 48 hours, GFP+ cells were sorted on the FACS DIVA (Becton Dickinson). After allowing the cells to recover, clones were selected by limiting dilution and diluted to single cells in 96-well plates. After clonal expansion, clones were screened by isolating genomic DNA with the QIAamp kit (Qiagen) and PCR of mouse exon 11. The primers were exon 11 forward 5′tggacgcaagaaagggataag3′ and exon 11 reverse 5′gctgtctgcttcctgactatg3′. PCR products were gel purified using the QIAQuick gel extraction kit (Qiagen). Purified products were first sequenced by Sanger (MACLAB). Of 14 surviving clones, 2 were positive for mutations in mouse exon 11.

### Western immunoblotting

Antibodies against alpha-tubulin (ab15246) and KLF2 (ab139699) were obtained from Abcam (Cambridge, MA). Antibodies against acetylated-alpha-tubulin (sc23950) were obtained from Santa Cruz (Dallas, TX). Actin (A2066) antibodies were obtained from Sigma-Aldrich (St. Louis, MO). Procedures were performed as previously described^16^.

### Bioelectric measurement

Nasal potential difference (NPD) measurements were obtained as previously described with modifications^30^. Mice were anesthetized using a ketamine/xylazine rodent cocktail given intraperitoneally, dosed at 0.012 ml/g mouse weight until plane of anesthesia is reached approximately 10 minutes later.

### Growth analysis

All mice were weighed every 5 days from 10–40 days old and then every 10 days thereafter. Only mice surviving until 100 days were included in growth curve analysis. Length was assessed by measuring mice from the tip of the nose to the base of the tail.

### MRI adipose tissue biodistribution

Relaxation-Compensated Fat Fraction (RCFF) MRI was used to provide quantitative *in vivo* assessments of subcutaneous and peritoneal adipose tissue volumes^31^. Briefly, each animal was anesthetized in 1–2% isoflurane and positioned in a 7 T Bruker Biospec (Bruker Inc., Billerica, MA) MRI scanner. The respiration rate (60 ± 20 breaths/minute) and core body temperature (35 ± 1 °C) were controlled throughout the imaging experiment. The RCFF-MRI acquisition was then used to generate high resolution coronal MRI images of the entire mouse at different echo times. All images were then exported for offline processing in Matlab (The Mathworks, Natick, MA). The RCFF-MRI image analysis method first generates separate fat and water images sets and then mathematically recombines these fat and water images to calculate fat fraction images [fat/(water + fat)] compensated for both T1 and T2 relaxation times. These quantitative fat fraction images provide the basis for automatic segmentation of adipose tissue (fat fraction >0.8) from other tissues such as liver and muscle. Subsequent segmentation of the peritoneal wall calculates peritoneal and subcutaneous adipose tissue volumes.

### Quantitative PCR

To evaluate gene expression of *Igf1*, RNA was isolated from liver by use of TRIzol (Invitrogen). One microgram of RNA was reversed transcribed into cDNA by use of QScript cDNA synthesis kit (VWR). Real-time quantitative PCR was performed on a StepOne PCR system (Applied Biosystems). Expression was assessed via TaqMan expression assays for *Igf1* (Mm00439560; Applied Biosystems). Expression was normalized to β-actin as the endogenous control. Each RNA sample was used to make cDNA in duplicate, and the expression results were then averaged to yield the final result. The average of each sample was then expressed as a percentage of expression from WT animals.

### Immunohistochemistry

Inguinal fat pads from adult (8–12 weeks) mice were used for immunohistochemical detection of periplipin A. Fat pads were removed and fixed in formalin for 24 h, washed in distilled water, and stored in 70% ethanol until sectioning. Fat was paraffin embedded and sectioned (7 *μ*m). Perilipin A was detected by immunofluorescence methods. Sections were deparaffinized in xylene and rehydrated through graded ethanol. Antigen retrieval was performed using 10 mM citrate buffer. Sections were blocked with 5% Donkey serum and 1%BSA for 1 hr. Tissue sections were incubated with primary antibody against Rabbit anti perilipin A (Ab3526, 1:500, Abcam, Cambridge, MA) overnight at 4 °C. After washing, secondary antibody conjugated to Alexa Fluor 594 (1:500, Invitrogen, Carlsbad, CA) was added for 2 hrs. Negative controls were run by omitting primary antibody. Nonspecific staining was not observed. Immunostained sections were cover slipped with Vectashield mounting medium with DAPI (Vector Laboratories, Burlingame, CA). Slides were visualized using Leica DM6000 microscope (20X objective) with Improvision’s Volocity software.

### Statistics

Statistical analysis was performed using analysis of variance (ANOVA) with Newman-Keuls post hoc test for comparison of multiple groups (3 or more groups). Fisher’s exact test was used for comparing survival and fertility of the *F508del*/*Hdac6* mice. P values of <0.05 were considered significant.

## Electronic supplementary material

Supplemental figures
