# Supplementary material for: Improved Growth Patterns in Cystic Fibrosis Mice after Loss of Histone Deacetylase 6
Source: Sci Rep. 2017 Jun 16;7:3676. doi: 10.1038/s41598-017-03931-2 (PMC5473831; doi:10.1038/s41598-017-03931-2)
Supplement: Supplementary file 1 — Supplemental figures [file 41598_2017_3931_MOESM1_ESM.pdf]

Supplemental information

IMPROVED GROWTH PATTERNS IN CYSTIC FIBROSIS MICE AFTER LOSS OF HISTONE DEACETYLASE 6

Sharon M. Rymut[a], Deborah A. Corey[a], Dana M. Valerio[a], Bernadette O. Erokwu[b], Chris A. Flask[a,b,c], Thomas J. Kelley[a,*], and Craig A. Hodges[a,d]

Full gels for figure 1.

Acetylated Alpha Tubulin (Ac-tub)

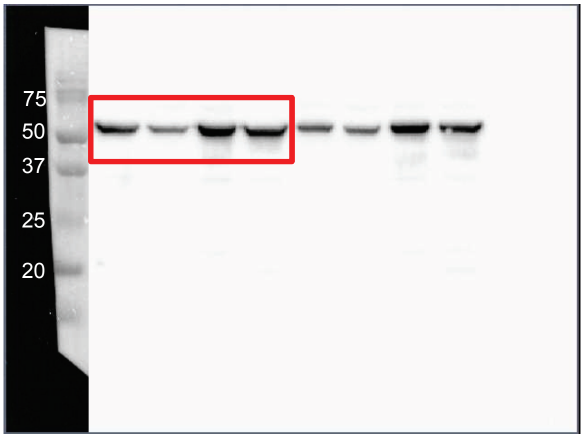

Lane 1: Standard
Lane 2: WT D28306
Lane 3: CF D28353
Lane 4: HDA HDA1125
Lane 5: CF/HDA HDA1161
Lane 6: WT D28325
Lane 7: CF D28398
Lane 8: HDA HDA1168
Lane 9: CF/HDA HDA1159

Alpha Tubulin

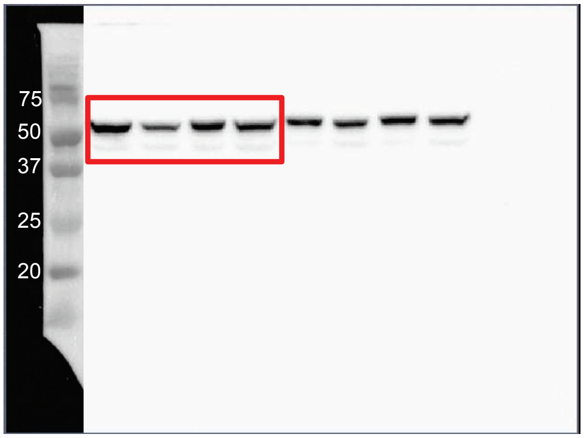

Lane 1: Standard
Lane 2: WT D28306
Lane 3: CF D28353
Lane 4: HDA HDA1125
Lane 5: CF/HDA HDA1161
Lane 6: WT D28325
Lane 7: CF D28398
Lane 8: HDA HDA1168
Lane 9: CF/HDA HDA1159

Actin

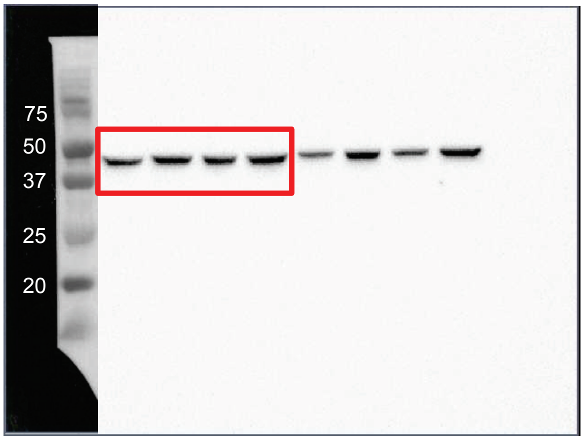

Lane 1: Standard
Lane 2: WT D28306
Lane 3: CF D28353
Lane 4: HDA HDA1125
Lane 5: CF/HDA HDA1161
Lane 6: WT D28325
Lane 7: CF D28398
Lane 8: HDA HDA1168
Lane 9: CF/HDA HDA1159

= edited gel for paper

Full gels for figure 5a

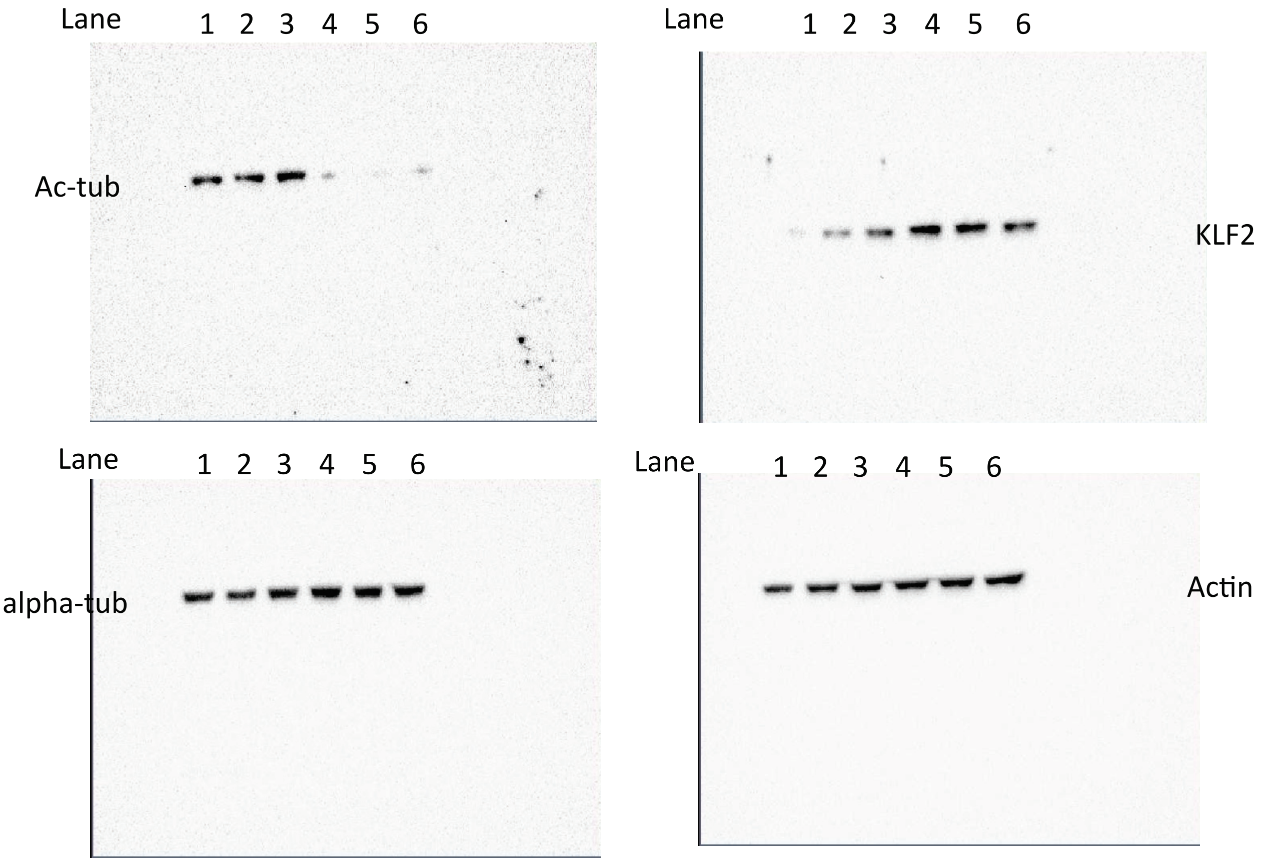

Lane 1,2, 3: WT 3T3-L1 cells
Lane 4,5,6: CF 3T3-L1 cells

Full gels for figure 5b

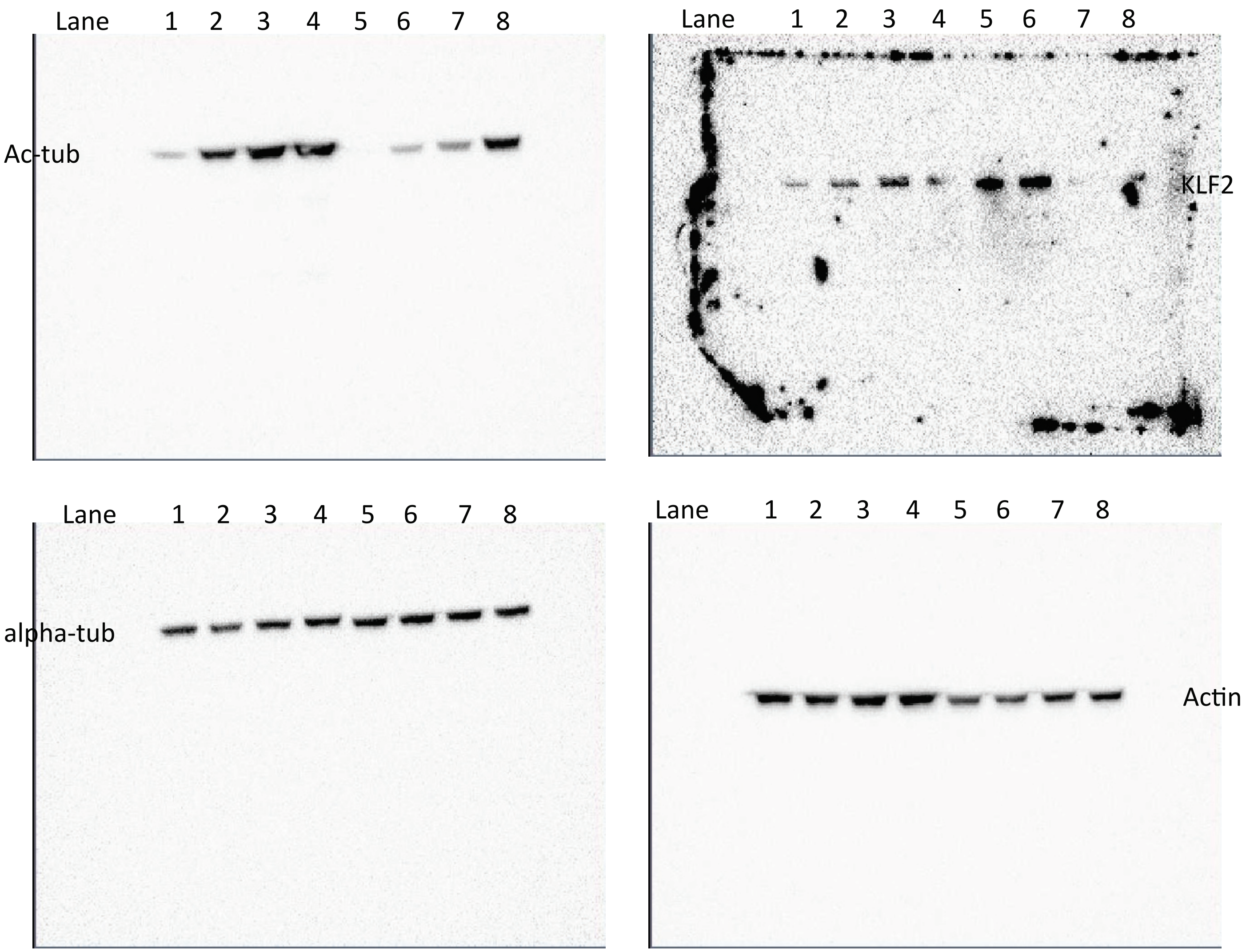

Lane 1: WT 3T3-L1 cells 0 µM tubastatin
Lane 2: WT 3T3-L1 cells 1 µM tubastatin
Lane 3: WT 3T3-L1 cells 5 µM tubastatin
Lane 4: WT 3T3-L1 cells 10 µM tubastatin
Lane 5: CF 3T3-L1 cells 0 µM tubastatin
Lane 6: CF 3T3-L1 cells 1 µM tubastatin
Lane 7: CF 3T3-L1 cells 5 µM tubastatin
Lane 8: CF 3T3-L1 cells 10 µM tubastatin

Full gels for figure 5c

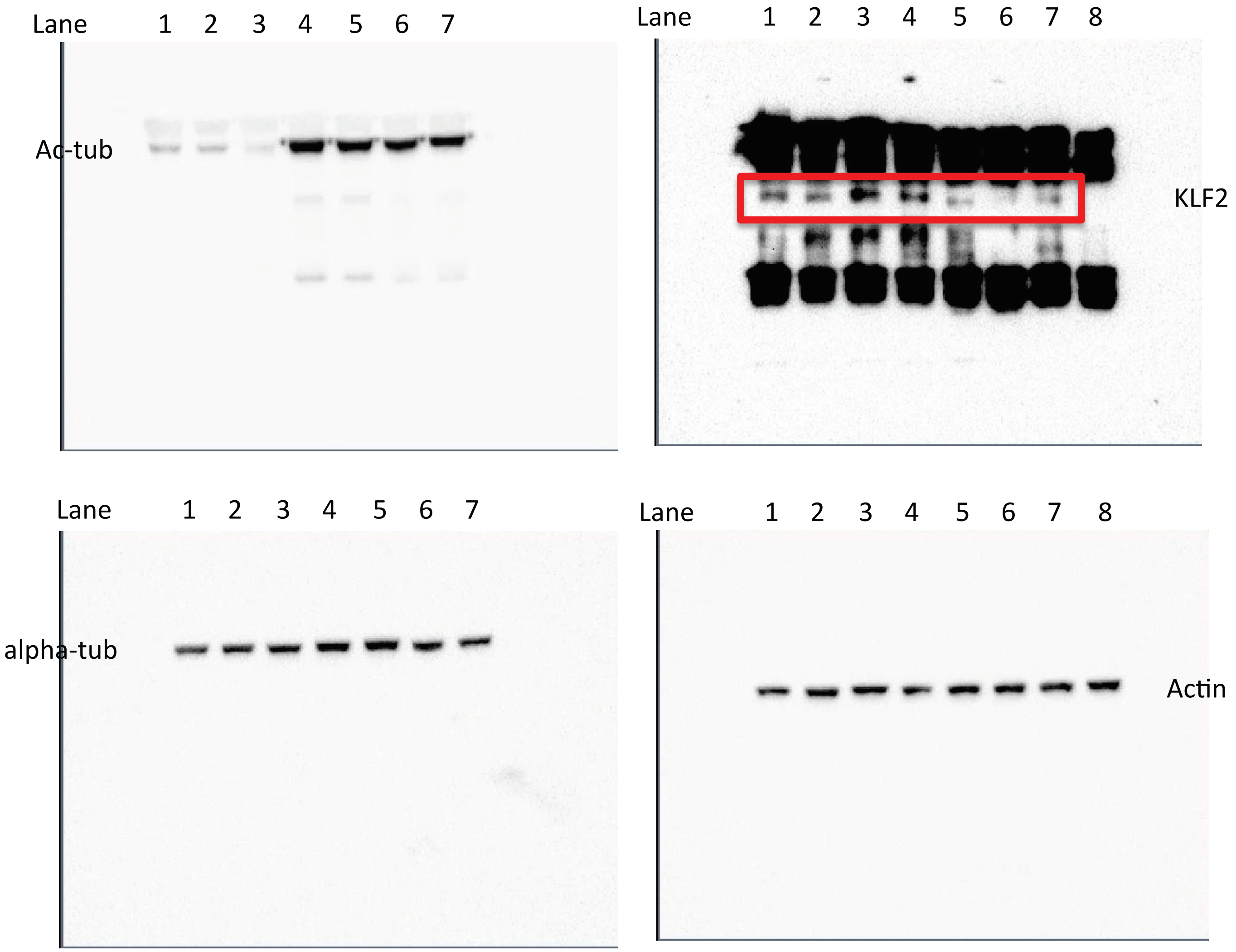

denotes cropping for figure

Lane 1: WT inguinal fat
Lane 2: WT inguinal fat
Lane 3: CF inguinal fat
Lane 4: HDA inguinal fat
Lane 5: HDA inguinal fat
Lane 6: CF/HDA inguinal fat
Lane 7: CF/HDA inguinal fat
Lane 8: CF/HDA inguinal fat (KLF2/actin gels)
